# Impacts of Sulfuric Acid on the Stability and Separation Performance of Polymeric PVDF-Based Membranes at Mild and High Concentrations: An Experimental Study

**DOI:** 10.3390/membranes10120375

**Published:** 2020-11-27

**Authors:** Kayode H. Lasisi, Weihao Yao, Temitope F. Ajibade, Huali Tian, Fang Fang, Kaisong Zhang

**Affiliations:** 1 Key Laboratory of Urban Pollutant Conversion, Institute of Urban Environment, Chinese Academy of Sciences, Xiamen 361021, China; lasisi@iue.ac.cn (K.H.L.); whyao@iue.ac.cn (W.Y.); temitope@iue.ac.cn (T.F.A.); hltian@iue.ac.cn (H.T.); ffang@iue.ac.cn (F.F.); 2 University of Chinese Academy of Sciences, Beijing 100049, China

**Keywords:** polyvinylidene fluoride, embedded membranes, sulfuric acid, stability, deterioration

## Abstract

This study investigated the effects of an aqueous acidic solution at typical concentrations on polymeric polyvinylidene fluoride (PVDF)-based membranes. Flat-sheet PVDF-based membranes were completely embedded in sulfuric acid at varying concentrations. The effect of the acid concentration after a prolonged exposure time on the chemical, mechanical and physical properties of the membrane were checked via FE-SEM, EDX (Energy-Dispersive Spectrometer), FTIR, XRD, tensile strength, zeta potential, contact angle, porosity, pure water flux measurement and visual observation. The result reveals prompt initiation of reaction between the PVDF membrane and sulfuric acid, even at a mild concentration. As the exposure time extends with increasing concentration, the change in chemical and mechanical properties become more pronounced, especially in the morphology, although this was not really noticeable in either the crystalline phase or the functional group analyses. The ultimate mechanical strength decreased from 46.18 ± 0.65 to 32.39 ± 0.22 MPa, while the hydrophilicity was enhanced due to enlargement of the pores. The flux at the highest concentration and exposure period increased by 2.3 times that of the neat membrane, while the BSA (Bovine Serum Albumin) rejection dropped by 55%. Similar to in an alkaline environment, the stability and performance of the PVDF-based membrane analyzed in this study manifested general deterioration.

## 1. Introduction

For several decades, membrane technology has proven efficient in the process of water treatment and separation techniques via the effective use of polymeric membrane materials [1,2,3,4,5,6,7]. These polymeric membranes have been reckoned to be cheaper and more efficient than other membranes because of their flexibility in spinning them into either spiral-wound or hollow-fiber modules. They have also displayed improved filtration performance and efficiency [8,9,10,11]. Most of the widely used commercial polymeric membranes materials for fabrication are polyimide (PI), polysulfone (PS), cellulose acetate (CA), polyamide (PA), polytetrafluoroethylene (PTFE), polypropylene (PP), polyvinylidene fluoride (PVDF) and polyethylene (PE). Out of these aforementioned materials, PVDF has held and still holds the greatest prospect in this class of polymers especially for both microfiltration (MF) and ultrafiltration (UF) applications owing to its numerous advantages [12,13,14]. As a semi-crystalline polymer having both a crystalline and amorphous phase, it exhibits high thermal stability, good mechanical properties, simple production flexibility and excellent chemical and oxidation resistances against strong acids, bases and oxidants [15,16,17,18,19,20,21]. In addition, selecting solvents for PVDF is effortless when compared with PTFE and PP, thus making it the preferred option in membrane applications [14,22]. One major setback of PVDF membranes, despite all their exciting characteristics, is their high hydrophobicity which stands as a great compromise between membrane selectivity and water flux, thus increasing fouling occurrences [10,23,24]. Several studies have been carried out to improve on this limitation via several methods such as surface modification/physical adsorption [25,26,27,28,29], hydrophilic polymer blending [30,31,32], plasma or chemical modification [33,34,35], graft polymerization [23,35,36], inorganic particle blending [37,38,39] and others. 

PVDF, as a versatile polymeric membrane, has been used both at laboratory and industrial scales under various conditions. Some of these applications include their use in mild or harsh environments via their exposure to chemicals which are harmful to the membrane materials and can alter their structure. This, after a prolonged period of operation, often leads to membrane degradation and a reduction in membrane stability [40,41]. Generally, more attention has been given to the overall system performance of PVDF membranes rather than checking the membranes’ material stability after long use in a harsh environment. Some studies have revealed the degradability and the performance level of these materials when pre-treated with organic or inorganic acids or bases during casting solution preparation or the coagulation process [42,43], while others have investigated the effect of these chemical solutions on the material properties and performance when post-treated or etched in either an acidic or alkaline environment under mild or severe conditions [44,45,46].

Ross et al. [47] examined the alkaline degradation of PVDF membranes by X-ray photoelectron spectroscopy, secondary ion mass spectrometry and Raman spectroscopy in order to determine the composition of the modified layer of the membrane using an alkali. The study revealed the formation of a surface layer consisting of bounded C=C bonds with the inclusion of oxygen functionalities in the treatment. Both the formation of C=C bonds and the presence of oxygen validated the defluorination and oxygenation which occurred during alkaline treatment. Puspitasari et al. [48] investigated the effect of sodium hypochlorite (NaClO) solutions on PVDF membranes in both the short- and long-term: there were chemical changes in the functional groups of the membrane, as revealed via Fourier transform infrared (FTIR) analysis, which thus significantly improved the membrane surface’s hydrophilicity. Abdullah and Bérubé [10] assessed the effect of extended NaClO exposure on PVDF-based micro-/ultra-filtration membranes at different concentrations for varying periods of time; they discovered that the period of the exposure had massive impact on the evolutions in the physical and chemical properties of the membrane than NaClO concentration. Hashim et al. [49] used and investigated the use of a sodium hydroxide (NaOH) solution and hydrofluoric (HF) acid as a post-treatment method for removing impurities from PVDF hollow-fiber membranes after formation. Experimental results showed that HF acid treatment was more effective at removing the impurities from the PVDF membrane compared with treatment with the NaOH solution. The mechanical properties of the hollow-fiber membrane treated with HF acid was found to be almost unaffected, while the one treated with the NaOH solution exhibited poor mechanical properties. In addition, Rabuni et al. [24] declared that exposure of a PVDF membrane to an alkaline solution caused some modification in the membrane’s texture, thus leading to structural degradation. This resulted from the chain splitting of the PVDF macromolecular skeleton via the dehydrofluorination reaction. They added that frequent chemical cleaning has an adverse effect on the membrane’s integrity and service life. Wang et al. [50] used analysis of variance to investigate the behavioral change of polyvinylidene fluoride (PVDF) membranes when cleaned with NaClO within a stipulated soaking period. The surface free energy and fouling propensity were also assessed. The analysis of variance showed that varying the NaClO soaking time had a significant effect on the membranes’ hydrophilicity and permeability. They further concluded that there was a reduction in the mechanical strength of the membrane as a result of NaClO chemical cleaning, which had a potentially harmful impact on the membrane’s stability, thus supporting the assertion of Rabuni et al. [24]. 

Despite the fact that some past studies have explored the effect of chemical exposure on the properties of PVDF-based membranes, most of the reports focused on the degradation mechanism in line with membrane stability and performance in an alkaline environment. Very limited studies have considered the effect in the acidic region. As summarized in the previous paragraph describing some of the previous work carried out, to the best of our knowledge, there has not been a detailed investigation on the impact of both mild and severe aqueous acidic concentrated solutions (either organic or inorganic acid) on the physicochemical and mechanical properties of flat-sheet polymeric PVDF-based membranes, subjected to an extended exposure period. However, it is worth mentioning that some earlier works were performed under extreme conditions in which high alkaline concentrations were used, either with or without a catalyst, for a longer exposure time. A systematic investigation of actual industry-related applications is still lacking, particularly in acidic conditions. In this study, we carefully investigate the effects of a sulfuric acid solution, both at mild and harsh concentrations, on PVDF membranes’ stability and performance at different exposure times. The terms mild and harsh are used based on the studies of Tanninen et al. [51] and Platt et al. [52]. This was further achieved via a morphology study, X-ray diffraction (XRD) analysis, Fourier transform infrared spectroscopy (FTIR) analysis, porosity, tortuosity, contact angle (hydrophilicity), zeta potential (surface charge), mechanical strength test, permeation flux and rejection studies. 

## 2. Experimental Conditions

### 2.1. Materials

A flat-sheet PVDF membrane with a molecular weight cut-off (MWCO) of 670,000–700,000 Da was kindly supplied by a renowned flat-sheet membrane manufacturer in Fujian Province, China. The preparation method for the PVDF membrane was not disclosed, as the supplier regarded this as proprietary information. The chemicals, including sulfuric acid (H_2_SO_4_, 98%), hydrochloric acid (HCl, 37%) and sodium hydroxide (NaOH, 100%), were all analytical grade, purchased from Beijing Chemical Co. (Beijing, China) and used as received for both the experiments and characterization. Bovine serum albumin (BSA) was obtained from Sigma-Aldrich (Selangor, Malaysia). All the washing and solution preparation was done using Millipore deionized (DI) water of 18 MΩ cm resistivity at 25 °C. 

### 2.2. Membrane Preparation and Embedding 

**Scheme 1:**
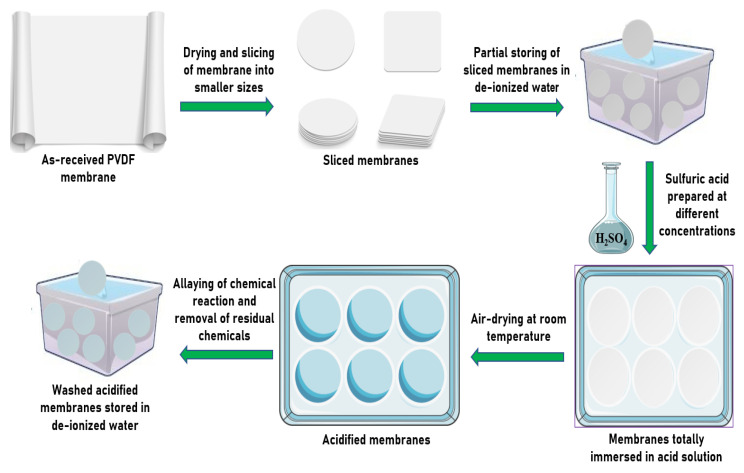
Embedding process of the as-received polyvinylidene fluoride (PVDF) membrane in an aqueous sulfuric acid solution.

The process of embedding membranes in sulfuric acid solution is shown in Scheme 1. In general, upon reception of the PVDF membrane in a rolled form, the membrane was initially gently spread out and thoroughly washed to remove any form of solvent impurities and preservatives. Thereafter, it was stored overnight in deionized water before the commencement of the experiment. The membrane was sliced into smaller sizes in order to allow for effective contact with the sulfuric acid solution when embedded. A sulfuric acid aqueous solution was prepared at five different concentrations (1.0, 2.0, 4.0, 8.0 and 15 mol/L) and allowed to cool down to room temperature before the embedding the sliced membranes. The periods of exposure were 7, 28, 56, 84 and 112 days. After the completion of each exposure period, the membranes were carefully rinsed and immersed in deionized water for 1 h in order to allay the reaction and also eliminate residual chemicals remaining on the membrane’s surface before checking the permeation flux and other characteristics. A pristine membrane was also assessed and characterized. For easy evaluation and explanation, the membranes are denoted as PVDF-neat and PVDF-*x*, where *x* represents 1, 2, 4, 8 and 15 for various concentrations of the acid solution at 1.0, 2.0, 4.0, 8.0 and 15 mol/L, respectively. 

### 2.3. Membrane Characterization

#### 2.3.1. Morphology 

The surface morphologies of the neat and embedded membranes were determined by a field emission scanning electron microscope (FE-SEM)(Hitachi S4800, Tokyo, Japan) with an accelerating voltage of 5.0 kV at various magnifications. Before the images were captured, the samples were positioned on a metal holder and sputtered with a thin layer of gold under a vacuum to reduce sample charging under the electron beam. The obtained FE-SEM images were then analyzed using ImageJ software (freely available, open-source software with a Java-based public domain image processing and analysis program) attached to the FE-SEM machine. A well-defined stepwise procedure of acquiring pore size data using ImageJ is provided in other studies [53,54]. In brief, the FE-SEM images were first adjusted and converted to 8-bit binary form by altering their brightness and contrast for better image resolution, and to achieve feasible image analysis. The binary image was then analyzed using the ‘analyze’ tool built into the software. Furthermore, the relative elemental compositions of the membrane surface were also obtained by mapping with an energy-dispersive spectrometer (EDX) attached to the FE-SEM machine.

#### 2.3.2. X-ray Diffractogram Analysis 

The crystalline phases and structural changes in both the neat and embedded membranes were obtained using an XRD-X’Pert PRO (PANalytical, The Netherlands). The membranes were gently placed on a thick plexiglass sample holder which was loaded on the diffractometer. The analysis was done using $CuK^{\propto }$ radiation (λ = 1.5418 Å), with the generator working at 40 kV and 40 mA. The scanning was performed within the range of 5 < 2θ < 90 with a scanning step of 0.02°.

#### 2.3.3. Fourier Transform Infrared Spectroscopy Analysis 

The Fourier transform infrared spectroscopy (FTIR) (Nicolet 6700 FTIR, Thermo Fisher Scientific Inc., Waltham, MA, USA) was used to qualitatively investigate the functional groups on the neat and embedded membrane surfaces with a resolution of 4 cm^−1^ and 32 scans per spectrum within a wave number range of 400–4000 cm^−1^. Prior to analysis, all samples were dried at room temperature for 24 h. The spectra were collected and processed using OMNIC software with an attenuated reflectance unit.

#### 2.3.4. Thickness, Porosity and Tortuosity

The thickness of the membranes was measured using a digital micrometer (293–252, Mitutoyo, Kanagawa, Japan, with a precision of ±0.01 mm). Ten various points were randomly selected on each membrane for measurement, and their average values and standard deviation were reported.

The porosity of both the neat and embedded membranes ε (%) was determined using the gravimetric method of measurement, which is defined as the ratio of the volume of pores to the total volume of the membrane. The wet membranes to be tested were sliced into small 3 by 3 cm pieces and weighed on a digital weighing balance (Mettler Toledo ME204E, Columbus, OH, USA) with ±0.001 readability [28]. Before measurement, the superficial water on the membrane surfaces was gently mopped off. These membranes were kept in an oven for 24 h at 60 °C before measuring the dry weight. The porosity was estimated by Equation (1).

(1) $$\varepsilon \;\left(\%\right)=\frac{\left(W_{W}-W_{D}\right)/\rho_{W}}{\frac{W_{W}-W_{D}}{\rho_{W}}+\;\frac{W_{D}}{\rho_{P}}}\;\times 100$$

where $W_{W}$ is the weight of the wet membrane (g),$\;W_{D}$ is the weight of the dry membrane (g), $\rho_{W}$ the pure water density (0.998 g/cm^3^) and $\rho_{P}$ is the polymer density (1.780 g/cm^3^).

Membrane tortuosity τ is the intrinsic ability of a membrane to withstand harsh conditions. It has an inverse relationship with porosity and it is dimensionless [55]. It was calculated by Equation (2).

(2) $$\tau =\frac{{\left(2-\varepsilon \right)}^{2}}{\varepsilon }$$

#### 2.3.5. Static Contact Angle Measurement 

The surface hydrophilicity of both the neat and embedded membranes was measured using a contact angle analyzer (DSA100, KRUSS, Hamburg, Germany). Dry membranes were sliced into 1.5 by 1.5 cm pieces and each was taped onto 250 × 150 × 10 mm flexible plexiglass. Through capillary action, 3 μL of deionized water was dropped from a microsyringe with a stainless steel needle onto the membrane’s surface at room temperature. The contact angle was determined as the angle between the solid surface and a tangent to the curved surface of the drop. Five measurements at different points on each membrane surface were averaged to obtain an accurate contact angle.

#### 2.3.6. Zeta Potential 

The surface charges of both the neat and embedded membranes were tested three times (to get average values) using a streaming potential analyzer (SurPASS 3, Anton Paar, North Ryde, Australia) with a 0.010 mol/L KCl electrolyte solution. Before the zeta potential was determined, each membrane was sliced into two rectangular samples using the clamping cell with an adjustable gap of 10 × 20 mm. The pH value of 7.0 ± 0.5 was used as a flowing liquid to adjust the 0.05 mol/L HCl and NaOH solutions at a room temperature of 25 ± 1 °C; a gap height of about 100 μm was maintained.

#### 2.3.7. Mechanical Strength 

The ultimate tensile strength and elongation of both the neat and embedded membranes were tested using a Shimadzu universal tensile testing machine at a room temperature of 25 ± 1 °C with a loading velocity of 50 mm/min and gauge length of 100 mm. Before measurement, the membranes were sliced into 2 by 10 cm pieces. Three measurements were obtained for each membrane and averaged to obtain an accurate measurement. The deviation between the membrane values and their average was less than 10%. 

#### 2.3.8. Physical Observation and Examination 

The embedded membranes were checked and examined thoroughly after the completion of each immersion time for any sign of discoloration, blistering, surface attack and cracking.

### 2.4. Permeation and Rejection Studies 

**Figure 1:**
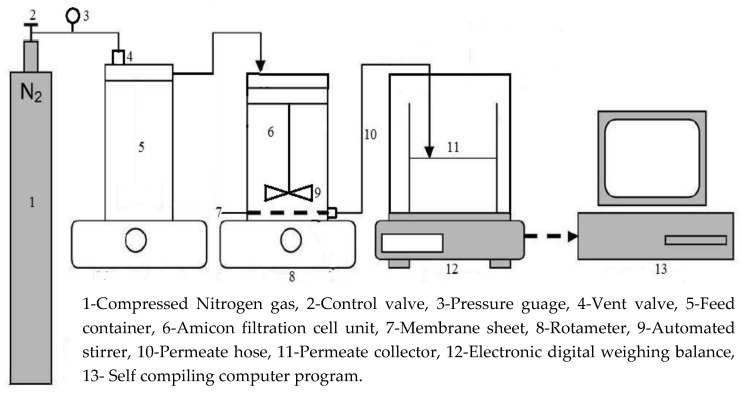
Schematic process of the dead-end filtration system setup.

The membrane permeation performance was measured using a self-designed laboratory scale in a dead-end filtration cell (Model 8010, Millipore Corp, Burlington, MA, USA) with an effective membrane area of 12.56 cm^2^. Figure 1 illustrates the schematic process of the filtration system. All filtration experiments were conducted under a nitrogen gas atmosphere and a stirring rate of 300 rpm to prevent deposition of contaminants at an ambient temperature of 25 ± 1 °C. Before each measurement was taken, the membranes were first pre-compacted using deionized water at a transmembrane pressure (TMP) of 0.15 MPa for 30 min to minimize compaction effects and acquire a stable flux. Thereafter, the test was carried out at TMP of 0.10 MPa and the pure water flux was measured for 10 min to achieve a constant value using a self-compiling computer program in real time. The permeation flux (*J*) in L/m^2^h was calculated by Equation (3).

(3) $$J=\frac{V}{A\Delta t}$$

where *V* is the volume of permeated water (L), *A* is the effective membrane area (m^2^) and ∆*t* is the permeation time (h).

The membrane’s separation performance was evaluated by feeding a BSA solution of 0.3 g/L into the filtration cell after testing the pure water flux of the membrane. The concentration of both the feed and permeate were determined using a UV-Vis spectrophotometer (Shimadzu UV-2450, Tokyo, Japan) at a wavelength of 280 nm. Two samples were tested for each membrane and the average value was recorded. The rejection capability (*R*) of the BSA was calculated by Equation (4).

(4) $$R\left(\%\right)=\left(1-\frac{C_{p}}{C_{f}}\right)\times 100$$

where $C_{p}$ and $C_{f}$ are the concentrations of both the permeate and the feed solutions, respectively.

## 3. Results and Discussion

### 3.1. Membrane Morphology and Elemental Composition 

**Figure 2:**
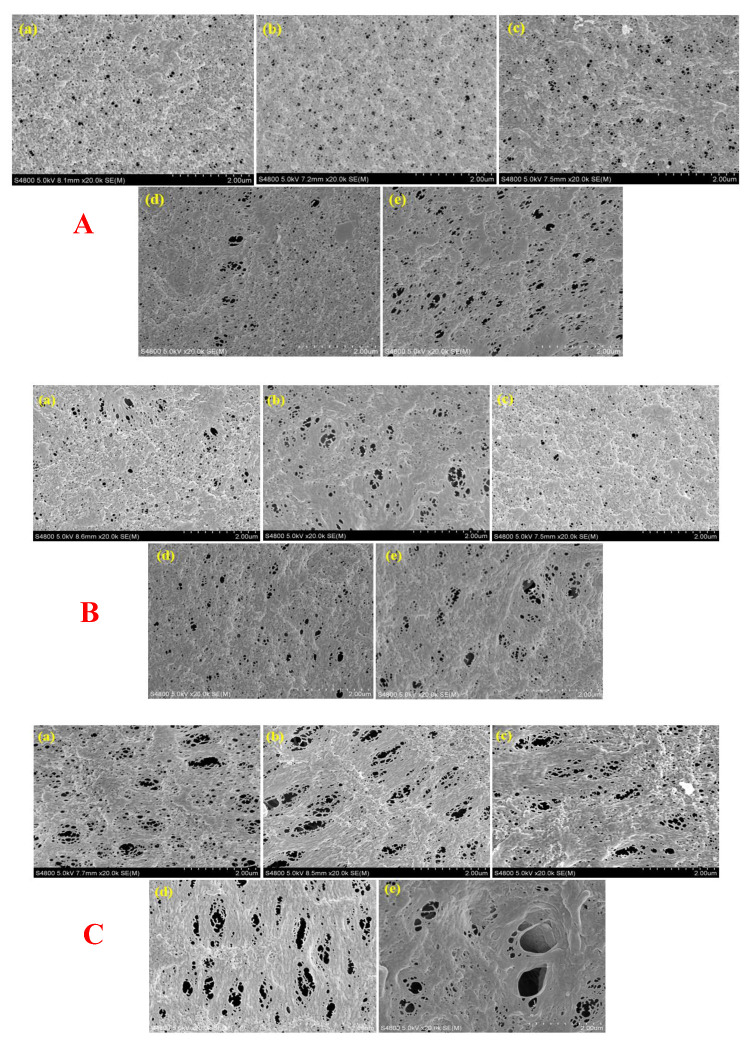

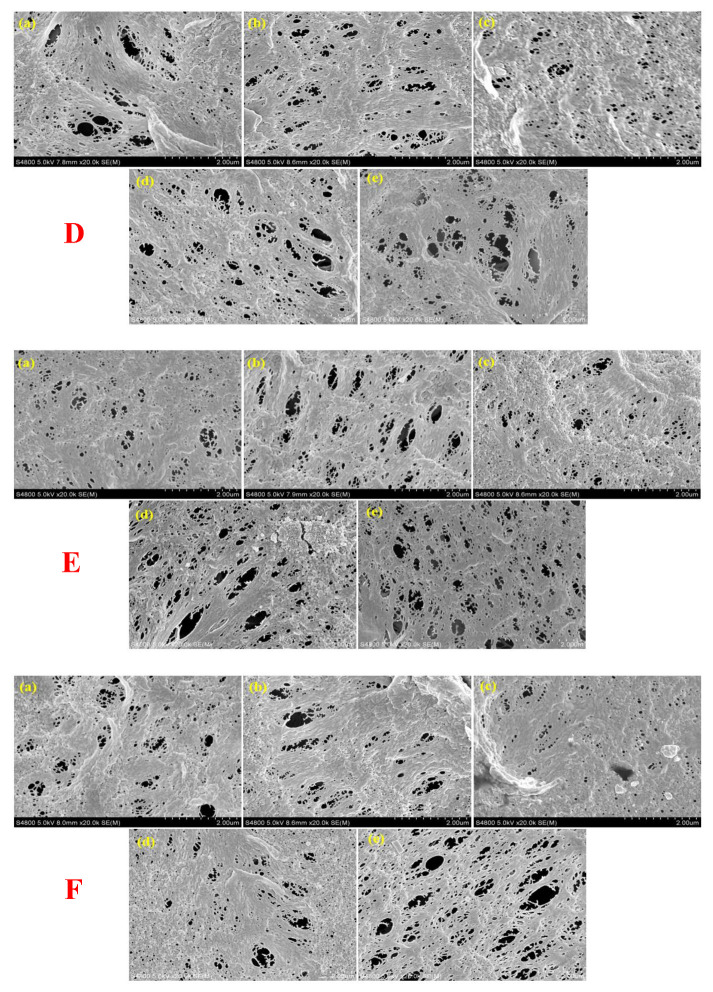
SEM images of the top surface of PVDF-Neat (**A**), PVDF-1 (**B**), PVDF-2 (**C**), PVDF-4 (**D**), PVDF-8 (**E**) and PVDF-15 (**F**) membranes on (**a**) Day 7, (**b**) Day 28, (**c**) Day 56, (**d**) Day 84 and (**e**) Day 112.

**Figure 3:**
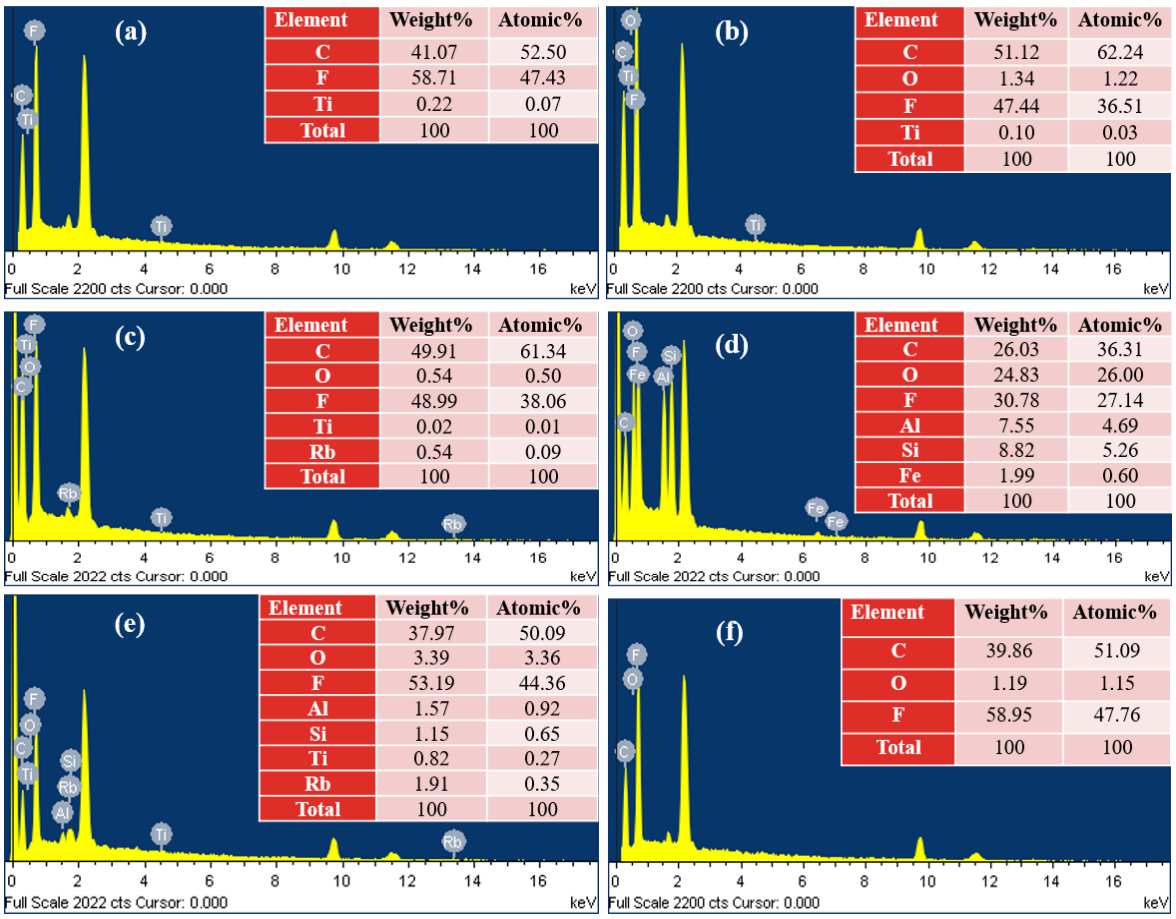
Elemental composition analysis of representative membrane samples: (**a**) PVDF-neat, (**b**) PVDF-1 on Day 7, (**c**) PVDF-2 on Day 28, (**d**) PVDF-4 on Day 56, (**e**) PVDF-8 on Day 84 and (**f**) PVDF-15 on Day 112.

To understand the influence of sulfuric acid on the membranes’ structure at various concentrations after the completion of each immersion day, the membranes’ top-section FE-SEM images were carefully examined at 20,000× magnification. The FE-SEM image of the neat membrane (stored in deionized water) was also captured at the completion of each exposure day to check if there were any significant changes in the membrane’s structure. Uniformly distributed pores, spread across the surface of the membrane, were more pronounced on exposure Day 7, 24 and 56, as shown in Figure 2 for A. For the embedded membranes, comparing the exposure time at varying acid concentrations in B–F, larger pores were observed on the membranes exposed to higher concentrations of acid than at lower concentrations. This largely influenced the porosity and permeated flux of the membranes, thus decreasing the hydrophobicity of the PVDF membranes. In addition, most of the acidified membranes at different concentrations and exposure times displayed several embossments (B–F), which were more severe on the surface of the PVDF-8 and PVDF-15 membranes. It is hypothesized that these pronounced embossments resulted from the chemical reaction occurring on the membranes’ surface caused by the acid concentration solution with a low pH. On the other hand, it should be noted that FE-SEM cross-section images of both the neat and embedded membranes are absent in this study because the nonwoven support of the PVDF membranes was extremely difficult to break, even in liquid nitrogen. Attempts made to view the few fractured layers under the SEM machine proved abortive. In addition, the pore size measurement was conducted using the known scale of the FE-SEM image in ImageJ. The average mean pore size of the PVDF-neat membranes was approximately 0.50 to 1.0 μm, while those of the embedded membranes ranged from approximately 1.25 to 2.0 μm. The elemental composition of representative membrane surfaces was further investigated by EDX analysis, as shown in Figure 3. It can be observed that compared with the neat membrane, a few metal contaminants such as Ti, Rb, Al, Ca and Si, which were not discovered in the neat membrane, were present in the embedded membranes at different concentrations. These were more evident in the PVDF-2 and PVDF-4 membranes on exposure Days 56 and 84, respectively, but these contaminants tended to diminish the longer the membranes stayed in the acid at higher concentrations. This observation could result from the prolonged exposure of the membrane to acid, which could lead to chemical reactions that present elemental impurities due to deterioration. Furthermore, these chemical reactions at different concentrations resulted in a loss of hydrogen and fluorine, which results in conjugation along the chains [24]. The main element found both in the neat and embedded membranes based on weight and atomic percentage composition is fluorine, followed by carbon, with a significant quantity of oxygen. This result is, however, expected, as the chemical composition of PVDF contains mostly carbon and fluorine [56,57]. 

### 3.2. XRD Analysis 

**Figure 4:**
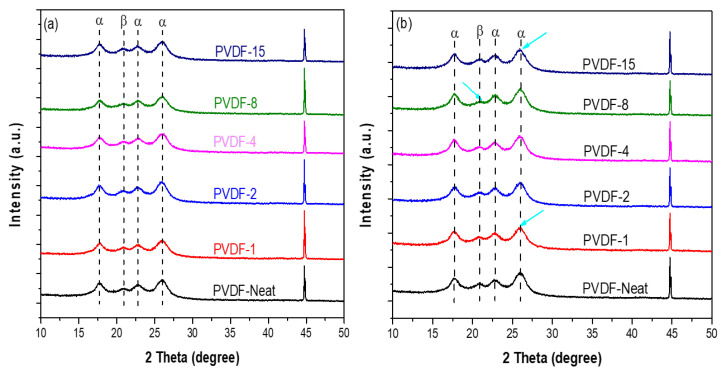
XRD crystalline phase analysis of neat and embedded membranes at exposure periods of (**a**) 7 days (representing the minimum exposure period) and (**b**) 112 days (representing the maximum exposure period).

To determine the alteration in the crystalline phase of the membranes embedded in different acid concentrations, XRD analysis was carried out. It has been established that PVDF polymers have five various crystalline forms known as the α, β, γ, δ and ε phases [56,58] but, according to previous findings, they are usually available as either the α or β phases and can periodically be present in the γ phase [59,60,61]. PVDF can also be systematically arranged in different crystal structures, depending on the processing of the material [62,63,64]. The peaks at diffraction angle 2θ is 18.01°, 20.01° and 26.58°, representing the diffractions in planes (020), (110) and (021) respectively. X-ray diffraction spectra collected for the embedded membranes at different exposure periods of 28, 56 and 84 days are provided in Figure S1, while Figure 4 shows the peaks for exposure Day 7 at different concentrations have approximately the same diffraction angles 2θ of 17.79, 20.76, 22.79, and 26.01 for the neat membrane, which correspond to the crystal planes of (100), (020), (110) and (021), respectively, representing the characteristic α phase of PVDF. The peak which appeared at 2θ = 20.76, originating from the crystal plane (110), reveals the existence of a β phase. In addition, a close observation of the characteristic 2θ peaks for PVDF-1, PVDF-2, PVDF 4, PVDF-8 and PVDF-15 are almost uniform with that of the neat membrane. Similarly, the peak for PVDF-neat at different concentrations on exposure Day 112 (shown in Figure 4) has diffraction angles 2θ of 17.72, 20.84, 22.79 and 25.99. These values reveal some slight differences when compared with the diffraction angles 2θ obtained for exposure Day 7. Although other membranes at different acid concentrations exhibit similar peaks to the neat membrane, some slight stretching was noticed, thus indicating the effect of the extreme conditions. Summarily, there were no serious changes detected in the crystal structure of the PVDF membranes, even at high concentrations of the sulfuric acid solution after a long period of exposure. Furthermore, all the spectra obtained for both the neat and acidified membranes were almost similar. This outcome shows that XRD analysis may be less effective at identifying changes made in the crystalline structure by sulfuric acid attack. A similar observation was made by [9] in the NaOH treatment of hollow-fiber PVDF membranes.

### 3.3. FTIR Spectroscopy 

**Figure 5:**
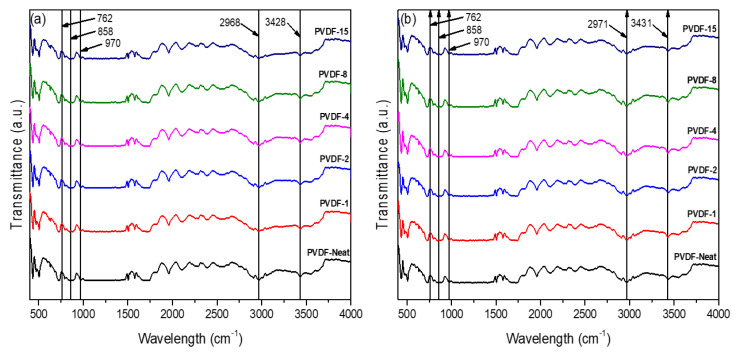
FTIR spectra of neat and embedded membranes at exposure periods of (**a**) 7 days (representing the minimum exposure period) and (**b**) 112 days (representing the maximum exposure period).

To analyze the chemical change in the functional group of the membranes, and to further validate the crystalline phase properties of the membrane surface layers obtained via XRD, attenuated total reflection Fourier transform infrared spectroscopy (ATR-FTIR) was applied. In addition, the effect of acid attack on the PVDF membranes at different concentrations, in terms of structural changes in the chemical groups, was analyzed. The FTIR spectra acquired for the PVDF-neat and embedded PVDF membranes at various concentrations (Figure 5a,b) generally show some of the common IR absorption bands at approximately 762, 858, 970, 1070 and 1149 cm^−1^, which is identical to the α phases of the PVDF crystal, while two other minor absorption bands noticed at 840 and 1404 cm^−^^1^ are envisaged as representing the β-phase. This is in line with the observations of some previously studied works [50,60,65]. In addition, the various absorption bands observed between 1450 and1950 cm^−1^ can likely be attributed to the dehydrofluorination occurring on the degraded membrane’s surface, causing the formation of more carbon–carbon double bonds (–CF = CH–), while the absorption bands between 2850 and 2950 cm^−1^ are attributed to stretched C–H bonds [9,57]. The degradation of PVDF membranes resulting in dehydrofluorination generally displayed a broad peak in the same region. Conjugated double bonds are also formed, which can aid the attachment of some specific reactants, thus introducing a functional group to the membrane [49]. There are some other noticeable absorption bands between 2950 and 3600 cm^−1^ in Figure 5a,b, with two particular pairs of bands at 2968 and 3428 cm^−1^, and 2971 and 3431 cm^−1^ for Days 7 and 112, respectively, representing the hydroxyl group. These peaks are associated with the action of oxidation or pre-treatment products such as sulfuric acid, nitric acid, carboxylic acids and alcohols [57,65,66,67]. The intensity of the hydroxyl peaks was observed to increase as the concentration of the acid present in the embedded membranes increased, on both exposure Days 7 and 112, although these changes were not visibly noticeable. This further proves the inertness and good chemical stability of PVDF membranes. Similar spectra were acquired for PVDF membranes embedded in various acid concentrations on exposure Days 28, 56 and 84, as shown in Figure S2. 

### 3.4. Membrane Thickness and Porosity

The thickness of a membrane gives insight into the flux and strength it may likely exhibit. Table 1 presents the thickness with the respective standard deviations of the neat and embedded membranes. It varies from 192.4 ± 3.95 to 196.4 ± 7.07 µm. Before the membranes were immersed in different acid concentrations, the thickness was measured to be 196.4 ± 7.07 µm at room temperature. This thickness was slightly higher than those of the embedded membranes, as they followed the decreasing order of PVDF-neat > PVDF-2 > PVDF-1 > PVDF-4 > PVDF-8 > PVDF-15. In addition, a sharp drop was noticed in the thickness of membrane PVDF-1 immediately after immersion in 1 mol/L acid concentration but was later justified by the corresponding thickness reduction in other membranes, with the exception of PVDF-2. This implies that the reduction in the membranes’ thickness with respect to different concentrations will increase the permeate flux, thus leading to the cost of higher heat loss and mechanical properties [20]. The porosity of the membranes was further checked using the dry and wet method. Table 1 shows that the porosity of the embedded membranes increased with an increase in acid concentration at the completion of each immersion period. A noticeable increment was observed in the porosity of the neat membranes after Day 7 of the exposure period, which indicates a sudden enlargement of the membrane’s pores, caused by acid attack on the membranes’ surface. However, some percentage variations were noticed in the porosity particularly for PVDF-1 and PVDF-2 on exposure Day 28; this is hypothesized to have resulted from a minute distortion in the membrane’s structure caused by the sudden acid attack on an inhomogeneous membrane spot. Moreover, slight and gradual increments were seen in the membrane porosity on other exposure days as the concentration of acid increased, but these were not as pronounced as on exposure Day 7. The overall porosity of the neat and embedded membranes ranged from 58.6 ± 0.42 to 73.2 ± 0.51%. This outcome further explains the SEM images in Figure 2, which reveal that the porosity changed with increasing acid concentration in most cases, as most of the pore sizes were seen to be visibly enlarged under the electron machine. 

### 3.5. Membranes’ Mechanical Strength and Tortuosity

**Figure 6:**
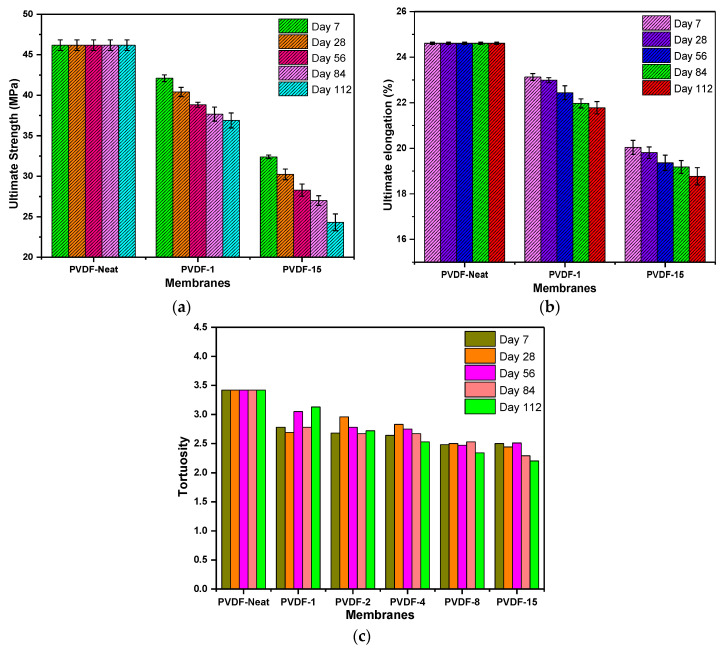
Mechanical properties of representative membrane samples: (**a**) ultimate strength of the membranes; (**b**) ultimate elongation of the membranes; (**c**) tortuosity of the membranes.

The lifespan of a membrane depends greatly on its mechanical strength. It has been reported that the properties of the polymer in use, in terms of membrane structure, morphology and degree of crystallinity, are imperative when assessing the mechanical properties of a membrane [24,68]. For an occasional assessment of the strength of a membrane, tensile strength and ultimate elongation are nonaggressive tests which can be adopted. In this work, these two parameters were measured to determine the effects of sulfuric acid on the membranes, at the lowest and highest concentrations and their respective exposure periods, to check the mechanical stability of the PVDF membranes. Those of the neat membranes were also checked for comparison and the results are shown in Figure 6a,b. The ultimate strength of the PVDF-neat membranes decreased from 46.18 ± 0.65 to 42.10 ± 0.41 and from 32.39 ± 0.22 MPa for PVDF-1 and PVDF-15, respectively, as they were in contact with the acid solution, and these reductions occurred gradually as the exposure days were extended. Similarly, the ultimate elongation, which represents the pertinacity of the membrane material, decreased in a stepwise manner from 24.61 ± 0.05% in the neat membrane to 21.78 ± 0.27% and 18.77 ± 0.38% for both PVDF-1 and PVDF-15 at the highest concentration and exposure period. As there was a decrease in the pertinacity of the membrane, this implies that the sulfuric acid caused some potential change in the membrane’s mechanical properties, especially at a high concentration, which could eventually lead to the mechanical instability of the membranes. Furthermore, the result obtained for porosity validated this outcome, as the mechanical properties of a membrane material do not depend only on the polymeric properties but also on the pore topology [68], although, these changes were not really noticeable in the XRD and FTIR analyses. The tortuosity of the membranes was mathematically derived through the membranes’ porosity. It is an intrinsic property which reveals the degree of the membranes’ ruggedness or twistedness when subjected to harsh conditions [55]. Figure 6c depicts the ruggedness of both the neat and embedded membranes. The results obtained have an inverse relationship with the membrane porosity. As expected, the tortuosity of the embedded membranes decreased gradually, both with increasing acid concentration and exposure period. In addition, the effects of both sulfuric acid concentration and exposure period were mild on the embedded membranes, as there were inconsiderable differences from the value obtained at the most extreme acidic conditions (i.e., PVDF-15) on Day 112 and the PVDF-neat membrane’s value. The overall tortuosity of the embedded membranes at all levels of concentration ranged from 2.20 to 2.96. Furthermore, the PVDF-8 and PVDF-15 membranes exhibited the least tendency to withstand extreme conditions, which affirms the weakening action taking place in the fiber of the membrane, caused by the acid reaction at extended concentrations and a longer exposure time. 

### 3.6. Membrane Hydrophilicity

**Figure 7:**
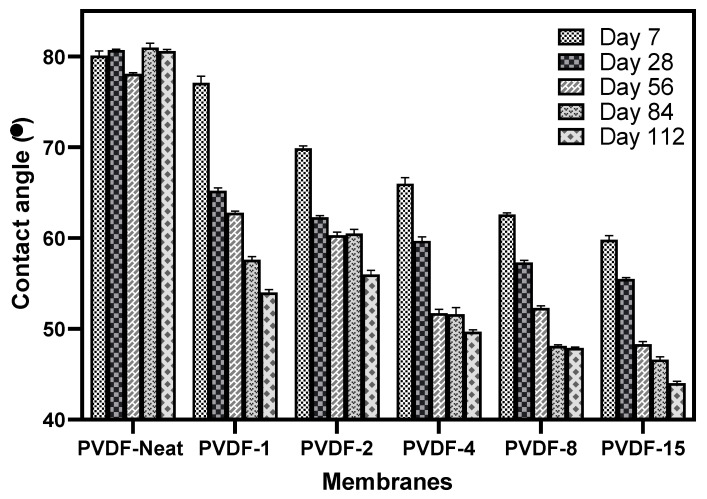
Contact angles of neat and embedded membranes at different acid concentrations.

To check alterations in the surface properties and also improve the antifouling property of membranes, the wetting ability of the membranes’ surface is a major determinant. This is often characterized through the contact angle measurement to check for either the hydrophobicity or hydrophilicity of the membrane [20]. The contact angles of the neat and embedded membranes are presented in Figure 7. The contact angle of the neat PVDF membrane was measured alongside all the embedded membranes on each day of testing. As seen from the result, the PVDF-neat membrane on all the days of immersion in deionized water presented the highest contact angle values, ranging from 78.1 to 81.0° with a minor difference of 2.9°. This outcome shows the hydrophobic nature of the PVDF membrane. The higher the contact angle of a membrane, the higher the hydrophobicity of its surface; the PVDF membrane, having a contact angle of 80 ± 5°, is categorized as hydrophobic [50]. However, a critical look at the result shows that sulfuric acid exposure has a great impact on the surface properties of the membrane. The PVDF-1 membrane has lower contact angle values than the neat membranes for all the days of immersion, thus exhibiting high hydrophilicity. Further exposure of the membranes to higher concentrations of 2 and 4 mol/L (PVDF-2 and PVDF-4) decreased the contact angle values from 69.9 to 56.0° and 66.0 to 49.7°, respectively, for all immersion and exposure periods. This thus further enhanced the hydrophilic nature of the PVDF membrane. In addition, with an extended exposure to sulfuric acid at concentrations of 8 and 15 mol/L, the contact angle followed the previous pattern by showing further reductions (thus increasing hydrophilicity) but with few differences. In general, the contact angle values of the PVDF membrane for all the acid concentrations considered, with the inclusion of the neat PVDF, decreased systematically with some insignificant exceptions for PVDF-2 and PVDF-4 on exposure Days 56 and 84, where the contact angle values were relatively close. The overall observed pattern is somewhat expected, as the SEM imagery revealed a similar pattern (Figure 2) by showing that the pore sizes of the membrane became larger in more concentrated acidic solutions, thus making the membranes’ surface more hydrophilic. 

### 3.7. Membrane Surface Charge

**Figure 8:**
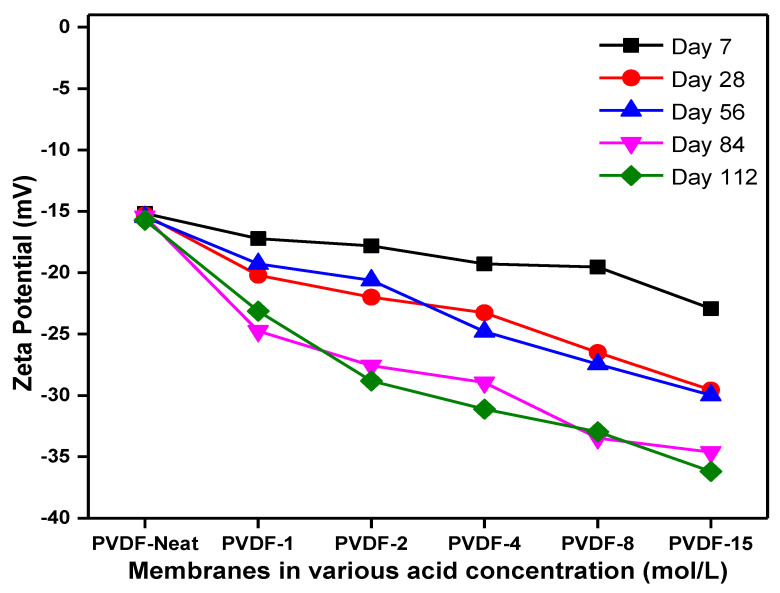
Surface zeta potential at different times of immersion of membranes embedded in varying sulfuric acid concentrations.

The charge present on the surface of a membrane is a major determinant in evaluating the antifouling performance of that membrane [69]. Zeta potential is widely used to provide vital information on the surface charge properties of a membrane. In this study, the zeta potential of the neat and embedded membranes was determined via the streaming potential method. The result presented in Figure 8 shows that all the membranes demonstrated negatively charged surfaces, with the values ranging from −15.17 to −36.19 mV. It was observed that the zeta potential of membranes calculated at low concentrations of acid were negative and became less negative as their concentration increased. This reduction in zeta potential with an increase in concentration could be explained by the protonation effect caused by the sulfuric acid attack on the membranes’ surface during the period of immersion. At higher concentrations (i.e., in a solution with a low pH value), there is the possibility of more contact ion pairs, which will not seriously contribute to the membranes’ surface charge, but when the concentration decreases, there is an increase in the absorption of cations; thus, a less negative surface charge is realized. Furthermore, a less negative zeta potential is observed for neutral surfaces tending to attract anions. The slight variation observed in the zeta potential of the neat PVDF membrane is speculated to be caused by some impurities that developed over time on the surface of the membrane stored in deionized water. The dependency of the antifouling properties of the membranes on both the charge on the membrane surface and that on the foulant under selected operational conditions has been established by [70]. In addition, the electrostatic interaction existing between the ions of the foulant in the feed solution and the charge on the membranes’ surface is an important factor, because when the zeta potential of a membrane is more negative, the tendency for fouling will reduce on the surface of that membrane; this is as a result of the electrostatic repulsion between the foulant in the feed solution and the membrane’s surface [45].

### 3.8. Physical/Visual Examination

**Figure 9:**
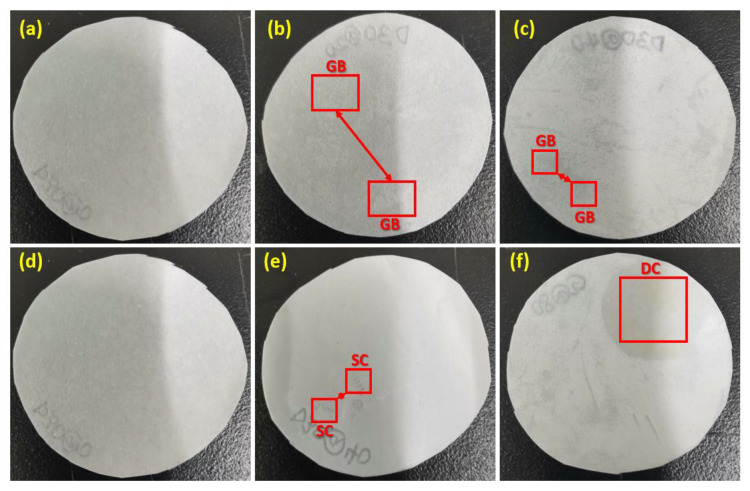
Physical inspection of the PVDF membranes at the end of exposure Day 7 for (**a**) neat membranes, (**b**) PVDF-1, (**c**) and PVDF-15, (**d**) and the end of Day 112 for neat membranes (**e**) PVDF-1 and (**f**) PVDF-15. Note: GB, SC and DC represent grouped blisters, slight cracks and discoloration, respectively.

The embedded membranes were inspected physically to observe if there were any changes in color. Two concentration boundaries (PVDF-1 and PVDF-15) of the said membranes were selected to represent the short- and long-term exposure conditions. Figure 9 depicts the visual appearance of the PVDF-neat, PVDF-1 and PVDF-15 membranes. No distinct change in color was observed between the neat membrane and the embedded ones, except that some grouped minor blistering and cracking was noticed on the surface of some of the embedded membranes at the end of exposure Day 7. On the contrary, there was a slight partial discoloration of the PVDF-15 membrane at the end of exposure Day 112, while some more pronounced blisters than those on exposure Day 7 were observed. Prior to immersion, the neat membrane was white in appearance but later changed slightly to light yellow after immersion (Figure 9f). It is noteworthy that despite the slight changes that occurred particularly on the surface of the membranes, they did not affect their chemical properties, as no serious stretches were observed in their peaks in Figure 4 and Figure 5. Furthermore, the temperature of the treating or immersing solution can influence the color change of membranes [40,56]. In this case, the temperature of the aqueous acid solution was set at 25 ± 2 °C, which partly contributed to the indistinct changes that occurred.

### 3.9. Membrane Water Permeability and BSA Rejection

**Figure 10:**
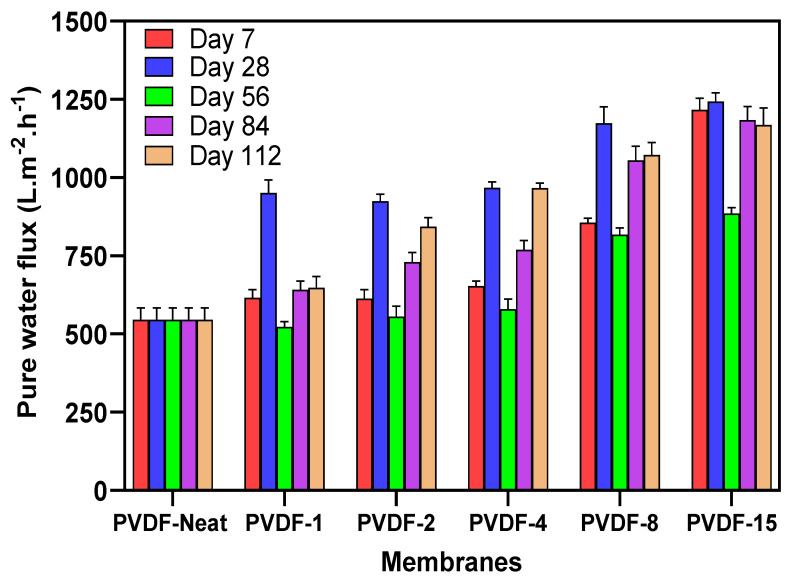
Permeate flux of neat and embedded membranes in varying sulfuric acid concentrations at different immersion periods.

It is generally noted that the pure water flux of a membrane is greatly influenced by its surface hydrophilicity, pore size and porosity [70,71]. From Table 1 and Figure 7, showing the porosity and contact angle results, respectively, it is seen that the hydrophilicity and the porosity of the embedded membranes gradually increased with increased acid concentrations but with a few exceptions. In the same manner, the permeation results in Figure 10 show a similar trend, as most of the membranes on exposure Days 7 and 28 displayed high fluxes, with an exceptional increase in membranes on exposure Day 28. This outcome indicates that the PVDF membranes’ properties were altered significantly, even with exposure to low acidic concentrations. The fluxes of the membranes exposed to 1 mol/L almost doubled after exposure Days 7 and 28, with a percentage increase of more than 90%, while these values almost tripled at 8 and 15 mol/L concentrations. This further increase could be explained by the protonation effect caused by the sulfuric acid attack on the membranes’ surface (which was earlier explained in Section 3.7), resulting in intense degradation of the membranes’ structure via pore enlargement. Meanwhile, an abrupt decrease was seen in the fluxes of all the membranes at all levels of acid concentration on exposure Day 56. This unusual reduction is hypothesized to be caused by the contamination of Al and Si resulting from the chemical reaction. However, there was a further increase in the water flux for all acid concentration levels as the days of exposure extended. This trend is akin to the FE-SEM images captured in Figure 2, which validated the anomaly in the results of the embedded membrane flux, as there was an enlargement of the pores in the membranes on exposure Days 7 and 28, which slightly shrank on exposure Day 56 and enlarged again on exposure Days 84 and 112. The neat membrane on all exposure days (although it was immersed in water) showed a flux with an average value of 545.5 Lm^−2^h^−1^. Generally, increases in the acidic or alkaline concentration and immersion time largely contribute to large flux increments, making the highest flux to be 1243.4 Lm^−2^h^−1^. In addition, the decrease in the contact angle values at higher concentrations of sulfuric acid, as seen in Figure 7, is a validation of the tuning of the PVDF membrane’s structure to become more hydrophilic. 

The BSA rejection ratios of the PVDF-neat, PVDF-1 and PVDF-15 membranes are presented in Table 2. These membranes are selected as representatives of the neutral, minimum and maximum values for the three identified membranes. The neat membrane (which is the same for all the exposure periods) gives the best rejection performance of 93.2%. Meanwhile, the rejection performance of the membranes decreased after their exposure to 1 mol/L concentration of the acid during the period of exposure. In addition, a further increase in the concentration of the acid up to 15 mol/L resulted in a more drastic reduction in the separation efficiency, with only the exception of membranes on exposure Day 56, which had better rejection performances for both the 1 and 15 mol/L concentration than exposure Days 84 and 112. The reduction in the rejection performance is envisaged to be caused by the alteration in the membrane’s surface morphology and properties due to deterioration. 

## 4. Conclusions

The effect of sulfuric acid on the chemical, mechanical and physical properties of a typical flat-sheet PVDF membrane, immersed at different concentrations and exposure times, was assessed using various analytical techniques. The outcome of the study reveals that the flat-sheet PVDF membranes were immediately attacked by the sulfuric acid aqueous solution, even at the lowest concentration. Further gradual deterioration in the properties of the membrane were observed at higher concentrations and exposure times. The morphology and pure water flux measurements revealed these gradual changes, which occur on the surface of the membrane. However, the changes in their crystalline phase and the functional group from the XRD and the FTIR analyses, respectively, were not significantly noticed, the decrease in the mechanical strength further attest to the fact that the membranes’ soundness was compromised. The zeta potential result shows the predominant charge on the immersed membranes, caused by the sulfuric acid attack, at both at low and high concentrations of acid, to be negative. In addition, the porosity, contact angle and visual examination results further affirm the impact of the acid on the PVDF membrane. The increase in the pure water flux and the corresponding decrease in the BSA rejection study show the inherent tendencies of deterioration which can occur on flat-sheet PVDF membranes which are subjected to use in an acidic environment.

## Figures and Tables

**Table 1:** Membrane porosity and thickness.

Membrane ID	Porosity (%)					Thickness (µm)	
	7 Days	28 Days	56 Days	84 Days	112 Days	Before	After
PVDF-neat	58.6 ± 0.42	58.6 ± 0.42	58.6 ± 0.42	58.6 ± 0.42	58.6 ± 0.42	196.4 ± 7.07	196.4 ± 7.07
PVDF-1	65.3 ± 0.47	66.3 ± 0.46	62.3 ± 0.40	65.3 ± 0.47	61.4 ± 0.46	196.4 ± 7.07	195.2 ± 4.85
PVDF-2	66.5 ± 0.56	63.2 ± 0.28	65.3 ± 0.27	66.6 ± 0.24	65.9 ± 0.25	196.4 ± 7.07	195.7 ± 5.19
PVDF-4	67.0 ± 0.13	64.7 ± 0.16	65.7 ± 0.52	66.6 ± 0.29	68.5 ± 0.43	196.4 ± 7.07	193.4 ± 2.55
PVDF-8	69.1 ± 0.20	68.9 ± 0.10	69.3 ± 0.18	68.4 ± 0.19	71.0 ± 0.16	196.4 ± 7.07	192.9 ± 3.42
PVDF-15	68.8 ± 0.30	69.7 ± 0.28	68.7 ± 0.42	71.8 ± 0.33	73.2 ± 0.51	196.4 ± 7.07	192.4 ± 3.95

**Table 2:** Rejection study of neat and embedded PVDF membranes.

Membrane ID	BSA Rejection (%)				
	7 Days	28 Days	56 Days	84 Days	112 Days
PVDF-Neat	93.2 ± 0.78	93.2 ± 0.78	93.2 ± 0.78	93.2 ± 0.78	93.2 ± 0.78
PVDF-1	70.7 ± 1.63	65.95 ± 3.04	76.8 ± 1.98	55.7 ± 1.34	49.9 ± 1.77
PVDF-15	57.0 ± 1.60	55.9 ± 1.35	56.9 ± 1.84	45.8 ± 1.27	31.4 ± 2.55

## Supplementary Materials

The following are available online at https://www.mdpi.com/2077-0375/10/12/375/s1, Figure S1: X-ray diffraction spectra for neat and embedded membranes on exposure Days 28, 56 and 84. Figure S2: FTIR spectra of neat and embedded membranes on exposure Days 28, 56 and 84. Table S1: Diffraction angles 2θ of the characteristic peaks.

## References

[1] He Z., Mahmud S., Yang Y., Zhu L., Zhao Y., Zeng Q., Xiong Z., Zhao S. (2020). Polyvinylidene fluoride membrane functionalized with zero valent iron for highly efficient degradation of organic contaminants. Sep. Purif. Technol..

[2] Dong C., Dai Y., Jiang S., He G. (2017). Application of Mg(OH)2 nanoplatelets as pore former to prepare PVDF ultrafiltration membranes. J. Environ. Chem. Eng..

[3] Huang X., Wang W., Liu Y., Wang H., Zhang Z., Fan W., Li L. (2015). Treatment of oily waste water by PVP grafted PVDF ultrafiltration membranes. Chem. Eng. J..

[4] Pezeshk N., Rana D., Narbaitz R.M., Matsuura T. (2012). Novel modified PVDF ultrafiltration flat-sheet membranes. J. Membr. Sci..

[5] Li X.Y., Chu H.P. (2003). Membrane bioreactor for the drinking water treatment of polluted surface water supplies. Water Res..

[6] Van Der Bruggen B., Vandecasteele C., Van Gestel T., Doyen W., Leysen R. (2003). A review of pressure-driven membrane processes in wastewater treatment and drinking water production. Environ. Prog..

[7] Wang X.M., Li X.Y., Shih K. (2011). In situ embedment and growth of anhydrous and hydrated aluminum oxide particles on polyvinylidene fluoride (PVDF) membranes. J. Membr. Sci..

[8] Hashim N.A., Liu F., Li K. (2009). A simplified method for preparation of hydrophilic PVDF membranes from an amphiphilic graft copolymer. J. Membr. Sci..

[9] Awanis Hashim N., Liu Y., Li K. (2011). Stability of PVDF hollow fibre membranes in sodium hydroxide aqueous solution. Chem. Eng. Sci..

[10] Abdullah S.Z., Bérubé P.R. (2013). Assessing the effects of sodium hypochlorite exposure on the characteristics of PVDF based membranes. Water Res..

[11] Pendergast M.M., Hoek E.M.V. (2011). A review of water treatment membrane nanotechnologies. Energy Environ. Sci..

[12] Ahmad A.L., Ramli W.K.W. (2013). Hydrophobic PVDF membrane via two-stage soft coagulation bath system for Membrane Gas Absorption of CO_2_. Sep. Purif. Technol..

[13] Rajabzadeh S., Yoshimoto S., Teramoto M., Al-Marzouqi M., Matsuyama H. (2009). CO_2_ absorption by using PVDF hollow fiber membrane contactors with various membrane structures. Sep. Purif. Technol..

[14] Bei P., Liu H., Yao H., Jiao Y., Wang Y., Guo L. (2019). Preparation and Characterization of a PVDF Membrane Modified by an Ionic Liquid. Aust. J. Chem..

[15] Ryzhikh V., Tsarev D., Alentiev A., Yampolskii Y. (2015). A novel method for predictions of the gas permeation parameters of polymers on the basis of their chemical structure. J. Membr. Sci..

[16] Liu F., Abed M.R.M., Li K. (2011). Hydrophilic modification of P(VDF-co-CTFE) porous membranes. Chem. Eng. Sci..

[17] Liu F., Hashim N.A., Liu Y., Abed M.R.M., Li K. (2011). Progress in the production and modification of PVDF membranes. J. Membr. Sci..

[18] Kang G.-d., Cao Y.-m. (2014). Application and modification of poly(vinylidene fluoride) (PVDF) membranes—A review. J. Membr. Sci..

[19] Hassan A., Niazi M.B.K., Hussain A., Farrukh S., Ahmad T. (2018). Development of Anti-bacterial PVA/Starch Based Hydrogel Membrane for Wound Dressing. J. Polym. Environ..

[20] Ali I., Bamaga O.A., Gzara L., Bassyouni M., Abdel-Aziz M.H., Soliman M.F., Drioli E., Albeirutty M. (2018). Assessment of blend PVDF membranes, and the effect of polymer concentration and blend composition. Membranes.

[21] Alyarnezhad S., Marino T., Parsa J.B., Galiano F., Ursino C., Garcìa H., Puche M., Figoli A. (2020). Polyvinylidene fluoride-graphene oxide membranes for dye removal under visible light irradiation. Polymers.

[22] Hou D., Wang J., Sun X., Ji Z., Luan Z. (2012). Preparation and properties of PVDF composite hollow fiber membranes for desalination through direct contact membrane distillation. J. Membr. Sci..

[23] Hudaib B., Gomes V., Shi J., Zhou C., Liu Z. (2018). Poly (vinylidene fluoride)/polyaniline/MWCNT nanocomposite ultrafiltration membrane for natural organic matter removal. Sep. Purif. Technol..

[24] Rabuni M.F., Nik Sulaiman N.M., Awanis Hashim N. (2016). A systematic assessment method for the investigation of the PVDF membrane stability. Desalin. Water Treat..

[25] Kavitskaya A.A. (2005). Separation characteristics of charged ultrafiltration membranes modified with the anionic surfactant. Desalination.

[26] Che A.F., Nie F.Q., Huang X.D., Xu Z.K., Yao K. (2005). Acrylonitrile-based copolymer membranes containing reactive groups: Surface modification by the immobilization of biomacromolecules. Polymer.

[27] Xi Z.Y., Xu Y.Y., Zhu L.P., Wang Y., Zhu B.K. (2009). A facile method of surface modification for hydrophobic polymer membranes based on the adhesive behavior of poly(DOPA) and poly(dopamine). J. Membr. Sci..

[28] Sun C., Feng X. (2017). Enhancing the performance of PVDF membranes by hydrophilic surface modification via amine treatment. Sep. Purif. Technol..

[29] Ma Z., Lu X., Wu C., Gao Q., Zhao L., Zhang H., Liu Z. (2017). Functional surface modification of PVDF membrane for chemical pulse cleaning. J. Membr. Sci..

[30] Asatekin A., Kang S., Elimelech M., Mayes A.M. (2007). Anti-fouling ultrafiltration membranes containing polyacrylonitrile-graft-poly(ethylene oxide) comb copolymer additives. J. Membr. Sci..

[31] Hester J.F., Banerjee P., Mayes A.M. (1999). Preparation of Protein-Resistant Surfaces on Poly(vinylidene fluoride) Membranes via Surface Segregation. Macromolecules.

[32] Hester J.F., Mayes A.M. (2002). Design and performance of foul-resistant poly(vinylidene fluoride) membranes prepared in a single-step by surface segregation. J. Membr. Sci..

[33] Kull K.R., Steen M.L., Fisher E.R. (2005). Surface modification with nitrogen-containing plasmas to produce hydrophilic, low-fouling membranes. J. Membr. Sci..

[34] Steen M.L., Jordan A.C., Fisher E.R. (2002). Hydrophilic modification of polymeric membranes by low temperature H2O plasma treatment. J. Membr. Sci..

[35] Wang P., Tan K.L., Kang E.T., Neoh K.G. (2002). Plasma-induced immobilization of poly (ethylene glycol) onto poly (vinylidene fluoride) microporous membrane. J. Membr. Sci..

[36] Zhou M., Liu H., Kilduff J.E., Langer R., Anderson D.G., Belfort G. (2009). High-throughput membrane surface modification to control NOM fouling. Environ. Sci. Technol..

[37] Dai J., Xiao K., Dong H., Liao W., Tang X., Zhang Z., Cai S. (2016). Preparation of Al2O3/PU/PVDF composite membrane and performance comparison with PVDF membrane, PU/PVDF blending membrane, and Al2O3/PVDF hybrid membrane. Desalin. Water Treat..

[38] Zeng G., He Y., Yu Z., Zhan Y., Ma L., Zhang L. (2016). Preparation and characterization of a novel PVDF ultrafiltration membrane by blending with TiO 2 -HNTs nanocomposites. Appl. Surf. Sci..

[39] Xie W., Li J., Sun T., Shang W., Dong W., Li M., Sun F. (2018). Hydrophilic modification and anti-fouling properties of PVDF membrane via in situ nano-particle blending. Environ. Sci. Pollut. Res..

[40] Rabuni M.F., Nik Sulaiman N.M., Aroua M.K., Hashim N.A. (2013). Effects of alkaline environments at mild conditions on the stability of PVDF membrane: An experimental study. Ind. Eng. Chem. Res..

[41] Rabuni M.F., Nik Sulaiman N.M., Aroua M.K., Yern Chee C., Awanis Hashim N. (2015). Impact of in situ physical and chemical cleaning on PVDF membrane properties and performances. Chem. Eng. Sci..

[42] Al-Gharabli S., Mavukkandy M.O., Kujawa J., Nunes S.P., Arafat H.A. (2017). Activation of PVDF membranes through facile hydroxylation of the polymeric dope. J. Mater. Res..

[43] Zhang W., Shi Z., Zhang F., Liu X., Jin J., Jiang L. (2013). Superhydrophobic and superoleophilic PVDF membranes for effective separation of water-in-oil emulsions with high flux. Adv. Mater..

[44] Muthukumar K., Jacob Kaleekkal N., Lakshmi D.S., Srivastava S., Bajaj H. (2019). Tuning the morphology of PVDF membranes using inorganic clusters for oil/water separation. J. Appl. Polym. Sci..

[45] Wang Q., Wang Z., Wu Z. (2012). Effects of solvent compositions on physicochemical properties and anti-fouling ability of PVDF microfiltration membranes for wastewater treatment. Desalination.

[46] Samsure N.A., Hashim N.A., Nik Sulaiman N.M., Chee C.Y. (2016). Alkaline etching treatment of PVDF membrane for water filtration. RSC Adv..

[47] Ross G.J., Watts J.F., Hill M.P., Morrissey P. (2000). Surface modification of poly(vinylidene fluoride) by alkaline treatment: 1. The degradation mechanism. Polymer.

[48] Puspitasari V., Granville A., Le-Clech P., Chen V. (2010). Cleaning and ageing effect of sodium hypochlorite on polyvinylidene fluoride (PVDF) membrane. Sep. Purif. Technol..

[49] Hashim N.A., Liu Y., Li K. (2011). Preparation of PVDF hollow fiber membranes using SiO2particles: The effect of acid and alkali treatment on the membrane performances. Ind. Eng. Chem. Res..

[50] Wang Q., Zeng H., Wu Z., Cao J. (2018). Impact of sodium hypochlorite cleaning on the surface properties and performance of PVDF membranes. Appl. Surf. Sci..

[51] Tanninen J., Platt S., Weis A., Nyström M. (2004). Long-term acid resistance and selectivity of NF membranes in very acidic conditions. J. Membr. Sci..

[52] Platt S., Nyström M., Bottino A., Capannelli G. (2004). Stability of NF membranes under extreme acidic conditions. J. Membr. Sci..

[53] Sun W., Chen T., Chen C., Li J. (2007). A study on membrane morphology by digital image processing. J. Membr. Sci..

[54] Mazzoli A., Favoni O. (2012). Particle size, size distribution and morphological evaluation of airborne dust particles of diverse woods by Scanning Electron Microscopy and image processing program. Powder Technol..

[55] Pisani L. (2011). Simple Expression for the Tortuosity of Porous Media. Transp. Porous Media.

[56] Xiao T., Wang P., Yang X., Cai X., Lu J. (2015). Fabrication and characterization of novel asymmetric polyvinylidene fluoride (PVDF) membranes by the nonsolvent thermally induced phase separation (NTIPS) method for membrane distillation applications. J. Membr. Sci..

[57] Dzinun H., Othman M.H.D., Ismail A.F., Puteh M.H., Rahman M.A., Jaafar J. (2017). Stability study of PVDF/TiO2 dual layer hollow fibre membranes under long-term UV irradiation exposure. J. Water Process Eng..

[58] Bei P., Liu H., Yao H., Hu A., Sun Y., Guo L. (2019). Preparation and characterization of PVDF/CaCO3 composite membranes etched by hydrochloric acid. Environ. Sci. Pollut. Res..

[59] Cui Z., Hassankiadeh N.T., Lee S.Y., Lee J.M., Woo K.T., Sanguineti A., Arcella V., Lee Y.M., Drioli E. (2013). Poly(vinylidene fluoride) membrane preparation with an environmental diluent via thermally induced phase separation. J. Membr. Sci..

[60] Ma W., Zhang J., Wang X., Wang S. (2007). Effect of PMMA on crystallization behavior and hydrophilicity of poly(vinylidene fluoride)/poly(methyl methacrylate) blend prepared in semi-dilute solutions. Appl. Surf. Sci..

[61] Sun H., Magnuson Z., He W., Zhang W., Vardhan H., Han X., He G., Ma S. (2020). PEG@ZIF-8/PVDF Nanocomposite Membrane for Efficient Pervaporation Desulfurization via a Layer-by-Layer Technology. ACS Appl. Mater. Interfaces.

[62] Chang H.H., Chang L.K., Yang C.D., Lin D.J., Cheng L.P. (2016). Effect of polar rotation on the formation of porous poly(vinylidene fluoride) membranes by immersion precipitation in an alcohol bath. J. Membr. Sci..

[63] Tao M.m., Liu F., Ma B.r., Xue L.x. (2013). Effect of solvent power on PVDF membrane polymorphism during phase inversion. Desalination.

[64] Gregorio R. (2006). Determination of the α, β, and γ crystalline phases of poly(vinylidene fluoride) films prepared at different conditions. J. Appl. Polym. Sci..

[65] Antón E., Álvarez J.R., Palacio L., Prádanos P., Hernández A., Pihlajamäki A., Luque S. (2015). Ageing of polyethersulfone ultrafiltration membranes under long-term exposures to alkaline and acidic cleaning solutions. Chem. Eng. Sci..

[66] Cai C., Fan X., Han X., Li J., Vardhan H. (2020). Improved desulfurization performance of polyethyleneglycol membrane by incorporating metal organic framework CuBTC. Polymers.

[67] Arkhangelsky E., Kuzmenko D., Gitis N.V., Vinogradov M., Kuiry S., Gitis V. (2007). Hypochlorite cleaning causes degradation of polymer membranes. Tribol. Lett..

[68] Elele E., Shen Y., Tang J., Lei Q., Khusid B., Tkacik G., Carbrello C. (2019). Mechanical properties of polymeric microfiltration membranes. J. Membr. Sci..

[69] Xie H., Saito T., Hickner M.A. (2011). Zeta potential of ion-conductive membranes by streaming current measurements. Langmuir.

[70] Escobar I.C., Van Der Bruggen B. (2015). Microfiltration and ultrafiltration membrane science and technology. J. Appl. Polym. Sci..

[71] Xu F., Wei M., Zhang X., Song Y., Zhou W., Wang Y. (2019). How Pore Hydrophilicity Influences Water Permeability?. Research.

