# Supplementary material for: Impacts of Sulfuric Acid on the Stability and Separation Performance of Polymeric PVDF-Based Membranes at Mild and High Concentrations: An Experimental Study
Source: Membranes (Basel). 2020 Nov 27;10(12):375. doi: 10.3390/membranes10120375 (PMC7760507; doi:10.3390/membranes10120375)
Supplement: Supplementary file 1 [file membranes-10-00375-s001.pdf]

membranes

Supplementary Materials: Impacts of Sulfuric Acid on the Stability and Separation Performance of Polymeric PVDF-Based Membranes at Mild and High Concentrations: An Experimental Study

XRD Analysis

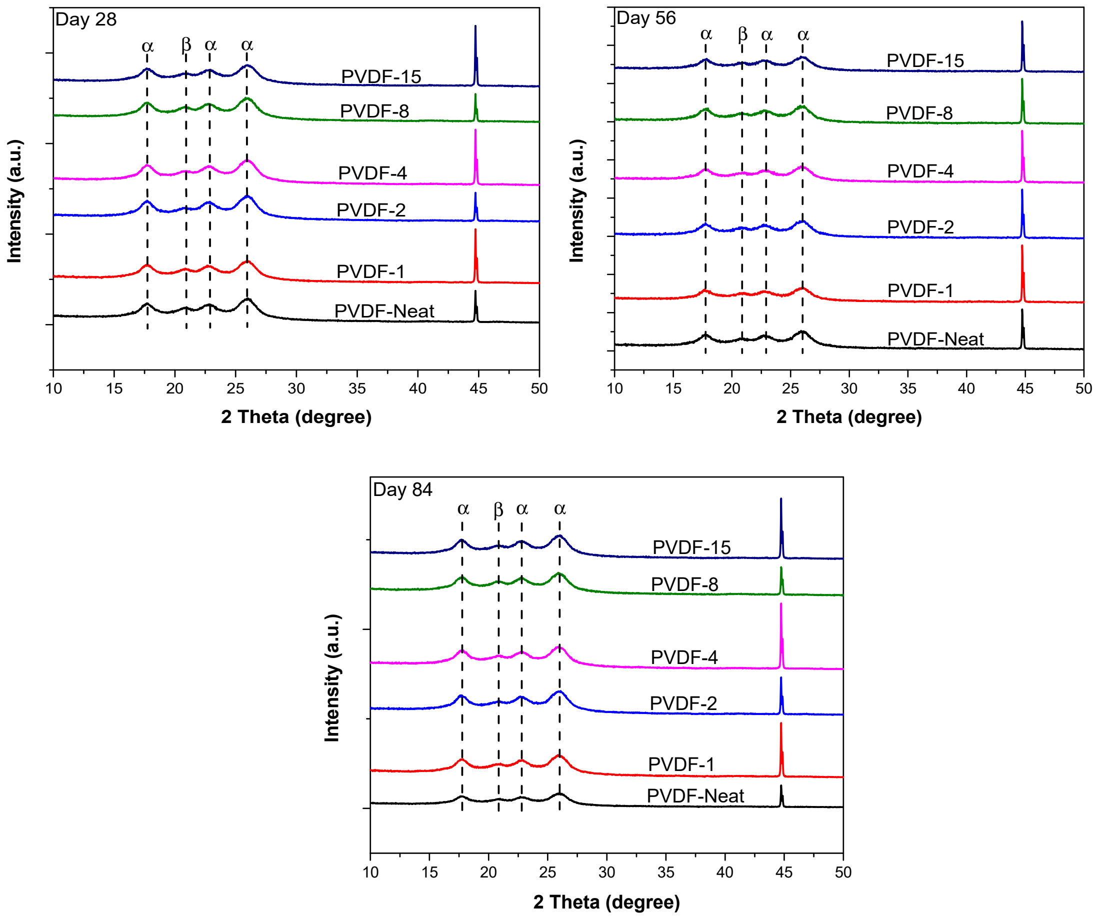

Figure S1. X-ray diffraction spectra for neat and embedded membranes at exposure day 28, 56 and 84.

Table S1. Diffraction angles 2θ of their characteristic peaks.

Exposure Period (Days)	Crystalline Phase Diffraction Angles 2θ			
	α	β	α	α
28	17.72	20.95	22.84	25.89
56	17.74	20.84	22.89	25.99
84	17.74	20.84	22.79	25.99

FTIR Analysis

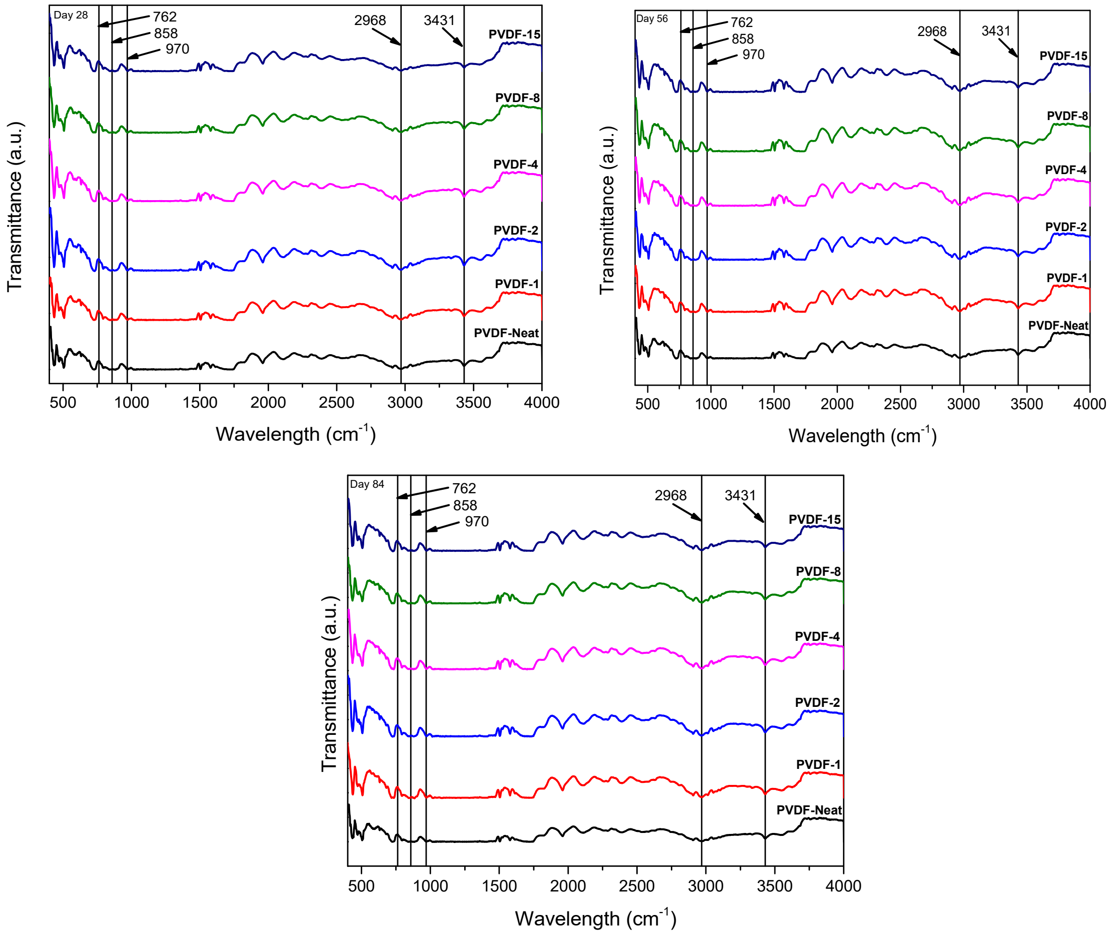

Figure S2. FTIR spectra of neat and embedded membranes at exposure day 28, 56 and 84.

© 2020 by the authors. Submitted for possible open access publication under the terms and conditions of the Creative Commons Attribution (CC BY) license (http://creativecommons.org/licenses/by/4.0/).
